# Feasibility and efficacy of mobile app implementation among patients with acute myocardial infarction enrolled in coordinated cardiac rehabilitation program

**DOI:** 10.3389/fdgth.2025.1606216

**Published:** 2025-11-06

**Authors:** Jan Harpula, Barbara Kalańska-Łukasik, Grzegorz Głód, Piotr Gąsierkiewicz, Oliwia Barnaś, Mateusz Danioł, Piotr Godek, Krystian Wita, Małgorzata Kowalska, Wojciech Wojakowski, Tomasz Jadczyk

**Affiliations:** 1 3rd Department of Cardiology, Faculty of Medical Sciences in Katowice, Medical University of Silesia, Katowice, Poland; 2 Department of Entrepreneurship and Management Innovation, University of Economics in Katowice, Katowice, Poland; 3 AllBright Technologies sp. z o.o., Kraków, Poland; 4 Department of Metrology and Electronics, Faculty of Electrical Engineering, Automatics, Computer Science and Biomedical Engineering, AGH University of Kraków, Kraków, Poland; 5 1st Department of Cardiology, Faculty of Medical Sciences in Katowice, Medical University of Silesia, Katowice, Poland; 6 Department of Epidemiology, Faculty of Medical Sciences in Katowice, Medical University of Silesia, Katowice, Poland; 7 Interventional Cardiac Electrophysiology Group, International Clinical Research Center, St. Anne’s University Hospital, Brno, Czechia; 8 Faculty of Space Technologies, AGH University of Kraków, Kraków, Poland

**Keywords:** mobile health application, cardiac rehabilitation, acute myocardial infarction, digital health intervention, telemedicine, patient adherence

## Abstract

**Introduction:**

Cardiovascular diseases (CVD), notably acute myocardial infarction (AMI), persist as a leading cause of global mortality despite advances in clinical management. Coordinated cardiac rehabilitation (CR) programs, such as the Coordinated Patient Care Program after Myocardial Infarction (MC-AMI), have demonstrated substantial reductions in mortality rates. However, optimizing outpatient care within these programs remains a challenge due to increasing patient volumes and physician workloads. This issue could be alleviated by using technology. Leveraging telemedicine solutions, particularly mobile apps, presents a promising avenue for addressing these challenges.

**Aim:**

The main objectives of this study were to determine if the dedicated mobile app for the cardiac rehabilitation program optimizes outpatient visit workflow and improves patient adherence to the CR program.

**Patients and methods:**

This observational study enrolled 103 patients after AMI, who completed the CR program and were eligible for the outpatient follow-up. Patients were divided into two groups: (1) the active group (*n* = 60) treated with a standard of care supplemented with the AHP-KOS app, and (2) the reference group (*n* = 43) treated with standard care without the AHP-KOS app. The first outpatient CR visit occurred 6 weeks after AMI.

**Results:**

Implementation of the AHP-KOS app was associated with higher adherence to the CR program (91.7% of patients using the mobile app completed 6-week outpatient visits vs. 67.4% of individuals treated with standards of care, *p* < 0.001). Additionally, the duration of onsite visits was significantly reduced in the active vs. reference group (8 ± 3 min. vs. 11 ± 4 min, *p* < 0.001, respectively).

**Conclusions:**

The AHP-KOS mobile app implemented in post-AMI resulted in higher adherence to the CR program (MC-AMI). Furthermore, the application of the AHP-KOS app resulted in financial and workflow optimization allowing for a significantly shorter time of outpatient visits.

## 1 Introduction

**Figure 1:**
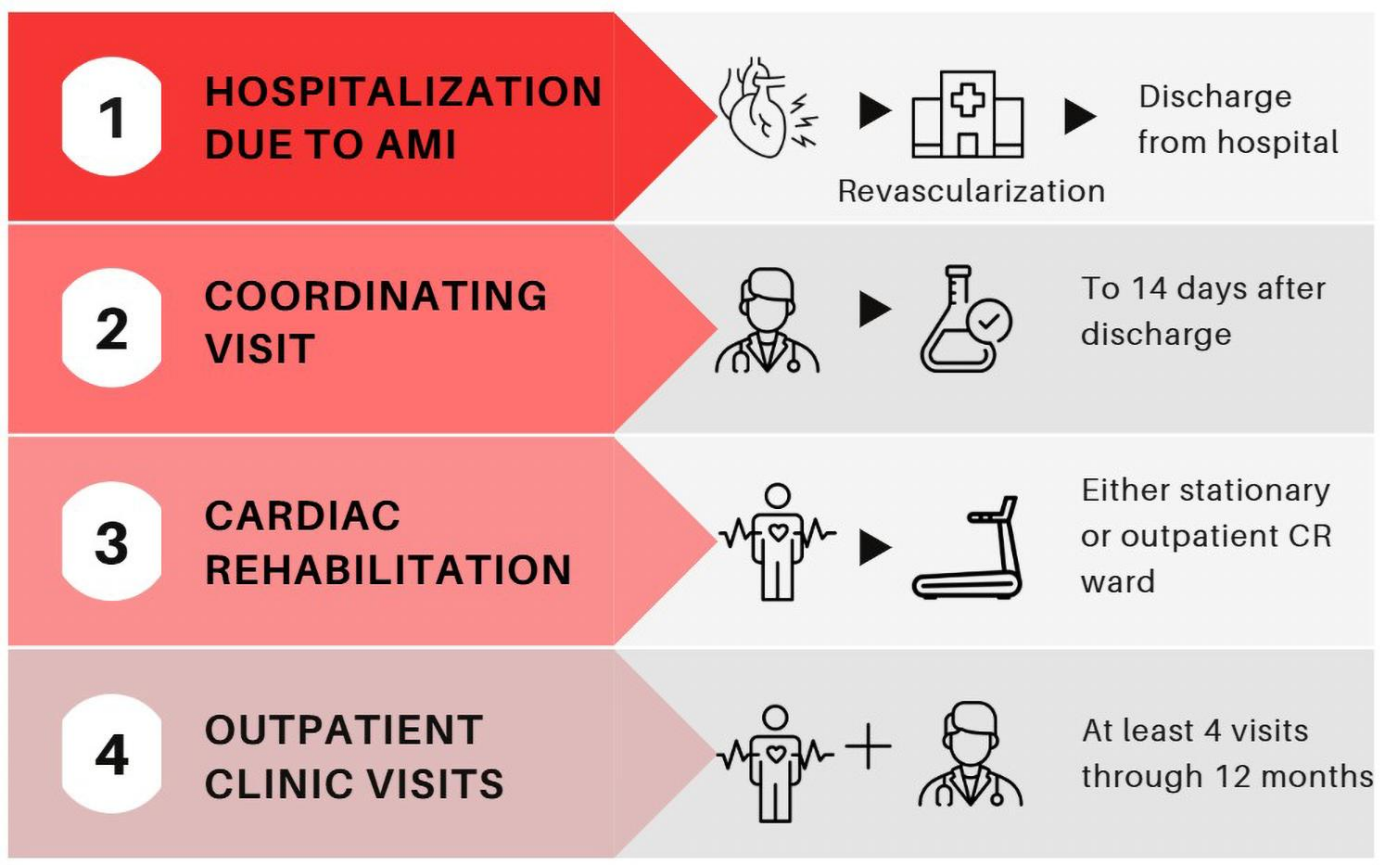
MC-AMI program overview. AMI, acute myocardial infarction; CR, cardiac rehabilitation.

Cardiovascular diseases (CVD) have the highest mortality among the global population with a coronary artery disease (CAD) being one of the most common causes of death (1, 2). Despite significant technological and pharmaceutical advancements, acute myocardial infarction (AMI) remains the most severe clinical presentation of CAD with an estimated annual toll of approximately 3 million lives (2, 3). Moreover, long-term mortality rates following AMI are significant reaching 25% within 5 years post-AMI (4–7). Due to clinical and social implications, the unfavorable outcomes indicated a need to implement coordinated cardiac rehabilitation (CR) programs in AMI patients. In 2017, the initiative entitled “Coordinated Patient Care Program after Myocardial Infarction” (MC-AMI) was piloted in several hospitals in Poland. Following the European Society of Cardiology guidelines, it was the first nationwide program regarding comprehensive care after myocardial infarction (8, 9). Figure 1 illustrates the MC-AMI program overview.

Current literature results have shown that participation in MC-AMI reduces 1-year mortality indicating that CR followed by outpatient care is the pivotal factors affecting clinical outcomes (10, 11). However, healthcare institutions experience excessive work burden since the volume of patients in the MC-AMI care continuously rises. Consequently, to provide high performance and quality of service, there is a need for optimization of the CR programs' workflows. CR programs face several operational challenges, including high patient volumes, limited availability of CR facilities, and staffing constraints. These factors often lead to extended wait times for appointments, reduced time for individualized patient care, and increased workload on healthcare providers. Additionally, logistical challenges such as patients' travel constraints, work schedules, and family responsibilities can interfere with their ability to attend regular CR sessions. To address these barriers, there is a need for workflow optimization in CR programs to ensure that services can be delivered efficiently without sacrificing quality. Mobile apps can facilitate CR workflow optimization features such as like pre-visit preparation, automated reminders or flexible scheduling (e.g., in the event of absence). A potential solution has become viable since mobile applications have been implemented in healthcare systems (12). According to the IBRIS (Polish Institute of Market and Social Research) “Seniors in the World of Mobile Applications” study at least 56% of people >70 years of age are using mobile apps, thus it may seem appropriate to presume patient-oriented mobile apps could help reduce cardiovascular risk burden (13, 14). Furthermore, due to the COVID-19 pandemic in recent years, the number of e-health and digital health solutions has increased, as well as the number of smartphone users, which could improve the feasibility of novel mobile app patient-enhancing programs (15, 16).

## 2 Aim

The main objectives of this study were to assess if the dedicated mobile app, AHP-KOS (1) optimizes outpatient visit workflow in MC-AMI patients, and (2) improves patient adherence to the CR program.

## 3 Methods

### 3.1 Application

Encouraged by the practical potential, we implemented the AHP-KOS® mobile app (AllBright Technologies, Warsaw, Poland) (17) exclusively developed for the MC-AMI program to test it on organizational workflow and patient adherence.

### 3.2 Patients

This observational study (performed 01.09.2022–31.01.2023) enrolled 103 AMI patients (mean age 66.7 years), who completed the CR program and were eligible for the outpatient follow-up. Recrutation process included patients who were admitted to our cardiology clinic with myocardial infarction, who at the time of discharge were asked if they want to participate in the cardiac rehabilitation program (mentioned before MC-AMI program). Therefore, for those who agreed were offered the eligibility to participate in a study in which they will use mobile app as an enhancement to the standard CR program.

However, as the active group membership required mobile app installation, the inclusion criteria besides informed consent were: basic digital literacy, ownership of smartphones with Android 5.0 or later/iOS 10.0 or later, internet accessability. Patients who weren't able to meet the inclusion criteria were transferred into the reference group.

Patients were divided into two groups: (1) Active group (*n* = 60, 58%) treated with a standard of care supplemented with the AHP-KOS app, and (2) reference group (*n* = 43, 42%) treated with a standard care without the AHP-KOS app.

Patients signed informed consent before the study started. The study obtained a local ethical committee agreement (PCN/CBN/0022/KB1/102/21, Ethical Committee of the Medical University of Silesia).

### 3.3 Onboarding process

Primarily, individuals with AMI were hospitalized at the 3rd Division of Cardiology, Medical University of Silesia. Study participants underwent percutaneous coronary intervention (PCI) with stent(s) implantation on the infarct-related artery.

Patients who joined the MC-AMI program were enrolled in the study at hospital discharge after signing the informed consent form. The AHP-KOS app sign-up process was assisted by the physician. In the first step, it was required to fill in personal data, provide the date of AMI and information about cardiovascular risk factors.

On the discharge, study coordinators assisted patients with correct app download and installation, registration on the patient profile, input of the basal information (medications) and were given full tutorial of the AHP-KOS app.

### 3.4 Overview of AHP-KOS mobile app and AllBright health platform

**Figure 2:**
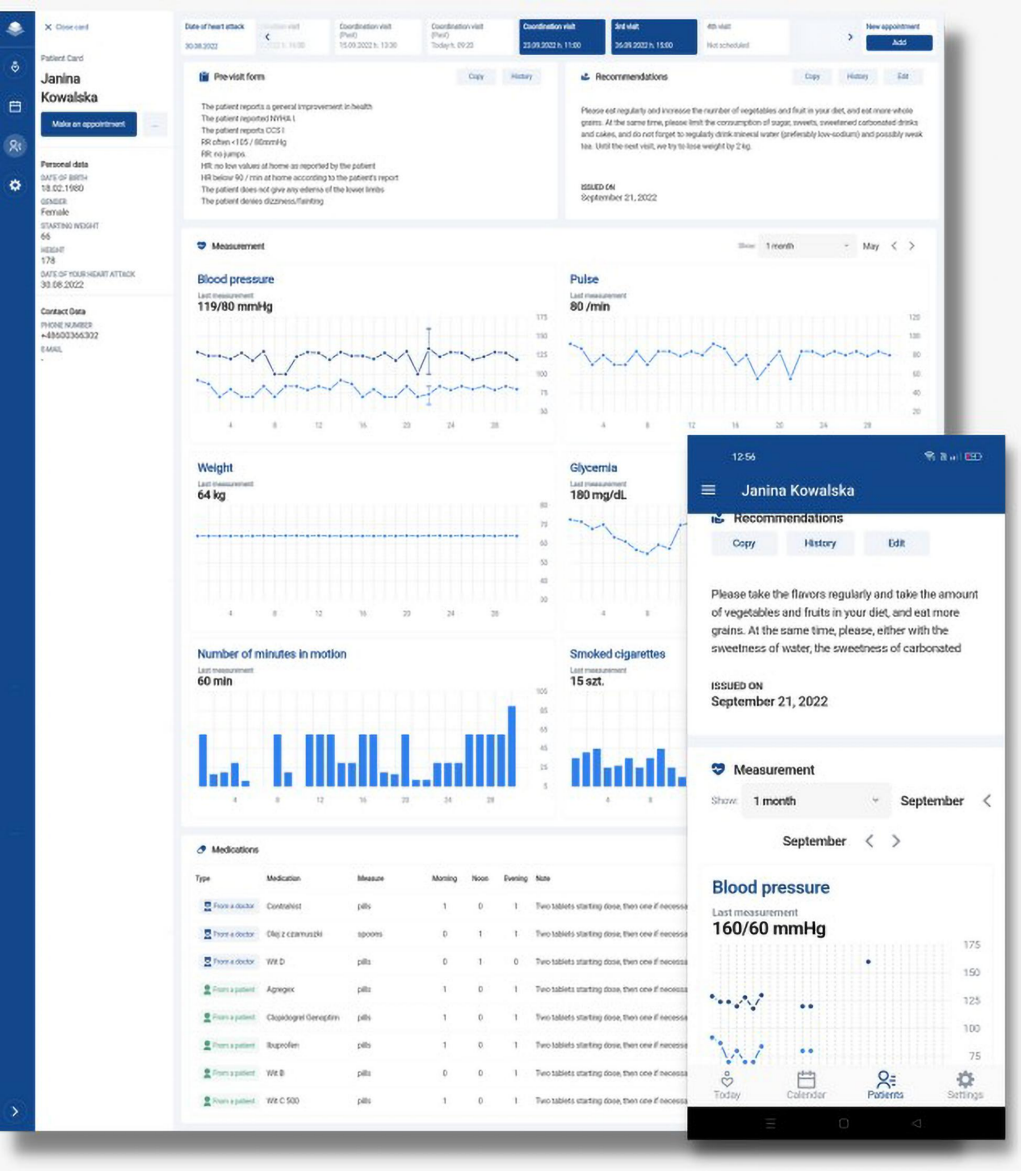
Overview of the AHP-KOS mobile app and AllBright Health Platform.

The integrated system allows for automatic data transfer of patient-reported outcome measures (PROMs) from the AHP-KOS mobile app to the AllBright Health Platform designed for the physicians and coordinators involved in the MC-AMI outpatient care (Figure 2). In accordance with the General Data Protection Regulation (GDPR), access to collected data was restricted to the authorized healthcare providers only. The system was constantly monitored in terms of stability, safety incidents, and adverse events.

#### 3.4.1 AHP-KOS mobile app

**Figure 3:**
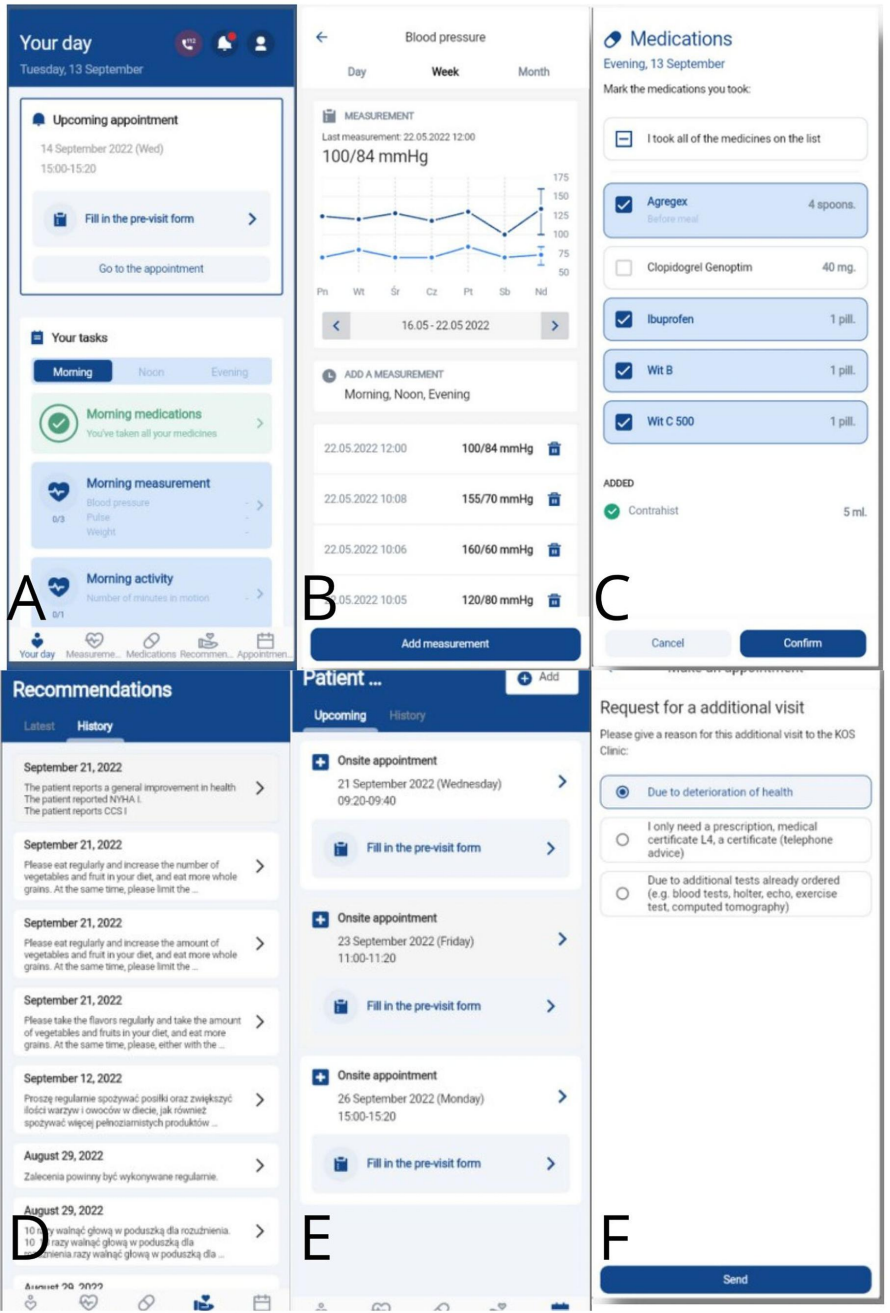
AHP-KOS mobile app user interface: **(A)** your day, **(B)** measurements, **(C)** medications, **(D)** recommendations, **(E)** visits, **(F)** request an appointment.

The AHP-KOS mobile app was downloaded from the Google Play Store (https://play.google.com/store/apps/details?id=health.allbright.kos) and installed on patients' smartphones (free for download). App interface consists of 5 core widgets: (A) Your day-medications reminders, measurements, and activities overview, (B) Measurements-PROMs including health data collected at home (blood pressure, heart rate, glucose level, and optionally blood test results), (C) Medications-summary of the pharmacological treatment recommended at discharge from the hospital including medication name, dose and number of intakes per day, (D) Recommendations-information for patients given at hospital discharge regarding e.g., body mass reduction and dietary guidelines, (E) Visits-information about upcoming outpatient visits with a possibility to reschedule the appointment. Furthermore, patients were able to inquire about an additional visit with an automatic notification sent to the MC-AMI coordinator who schedules the visit. Figure 3 illustrates the AHP-KOS application interface. The interventions that were able to perform by the app were the daily medications reminders with dosage information, as well as automatic reminders for vital signs assessment, the pre-visit questionnaire and contact option for the urgent situations.

#### 3.4.2 Pre-visit medical questionnaire

A few days before the planned MC-AMI outpatient visit, patients were notified to fill out an in-app medical questionnaire including 14 pre-specified clinical questions designed to assess symptom severity. Specifically, the survey contained questions regarding clinical status [angina pectoris severity with the chronic coronary syndromes (CCS) scale, subjective level of tiredness], vital signs (blood pressure, heart rate), symptoms (edemas, cough, vertigo, syncope, bleeding incidents), and overall wellbeing (Supplementary Material presents pre-visit medical questionnaire). After completion, PROMs were transferred to the physician via the AllBright Health Platform.

#### 3.4.3 AllBright health platform

**Figure 4:**
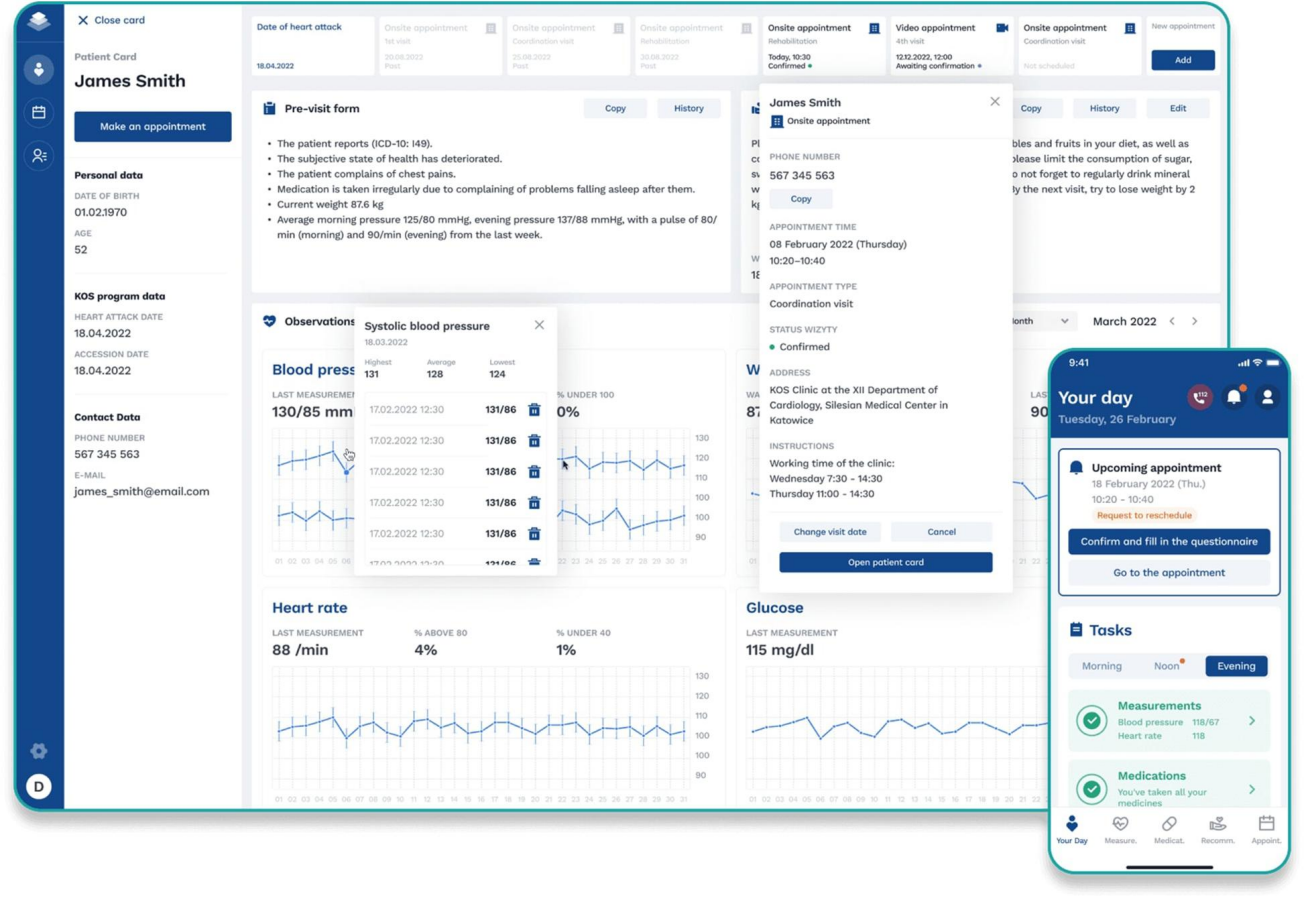
AllBright Health Platform—individual patient profile.

Through the AllBright Health Platform, cardiologists and coordinators involved in the outpatient MC-AMI program had access to (A) the main calendar visualizing scheduled visits for study participants and (B) individual patient profiles with home-obtained PROMs covering answers to pre-visit questionnaire, current medications (possibility to adjust and modify pharmacology with an automatic update of recommended drugs in the AHP-KOS app), as well as an option to schedule next visit. Figure 4 presents a basic view of the AllBright Health Platform.

## 4 Statistical analysis

Statistical analysis was performed with the use of the Statistica software (version 13.0; Dell Software Inc.). Qualitative variables such as sex, presence of a given disease, or medication taken were presented as a number and percentage of patients. Fishers test or chi-square test was used to assess differences between independent groups of patients. Differences in quantitative variables were assessed by the *U*-Mann–Whitney or by the *t*-Student test, depending on the distribution of particular variables assessed in the Shapiro–Wilk test. In the interpretation of results, *P* values < 0.05 or equal were considered statistically significant.

## 5 Results

Patients with the APH-KOS app (active group) were significantly younger than the control group. Furthermore, the groups differed significantly in terms of sex, occurrence of chronic kidney disease, peripheral vessel disease, and recieved angiotensin-converting enzyme inhibitors/angiotensin receptor blockers and statins therapy after discharge. The full characteristics of patients recruited from both groups are presented in Table 1.

**Table 1:** Baseline characteristics of the compared groups of patients.

Parameter	Active group (*n* = 60)	Reference group (*n* = 43)	*P* value
Age [years] (mean ± SD)	62.9 ± 8	70.5 ± 13	<0.01****
Female, *N* (%)	15 (25.0)	21 (48.8)	0.01**
Hypertension, *N* (%)	50 (83.3)	35 (81.4)	0.63**
Diabetes mellitus, *N* (%)	10 (16.7)	10 (23.3)	0.40**
Chronic kidney disease, *N* (%)	15 (25.0)	4 (9.3)	0.02*
Peripheral vessel disease, *N* (%)	4 (6.7)	9 (20.9)	0.03*
Atrial fibrillation, *N* (%)	4 (6.7)	4 (9.3)	0.91*
Implantable cardiac device (pacemaker, ICD, CRT-D), *N* (%)	2 (3.3)	1 (2.3)	0.78*
History of stroke, *N* (%)	5 (8.3)	3 (6.9)	0.79*
History of cardiac surgery, *N* (%)	6 (10.0)	6 (13.9)	0.53**
Glomerular filtration rate [ml/min/1.73 m^2^] (mean ± SD)	68.25 ± 23.57	78.61 ± 17.05	0.05***
Total cholesterol [mg/dl] (mean ± SD)	177.18 ± 44.43	167.32 ± 42.40	0.26****
LDL [mg/dl] (mean ± SD)	109.1 ± 42,59	106.4 ± 40.96	0.74****
HDL [mg/dl] (mean ± SD)	45.92 ± 17.04	41.19 ± 12.84	0.17***
Triglycerides [mg/dl] (mean ± SD)	134.72 ± 76.28	125.84 ± 58.24	0.84***
Pharmacotherapy			
Antiplatelet/Anticoagulation therapy			
ASA + clopidogrel, *N* (%)	43 (71.6)	34 (79.1)	0.46**
ASA + ticagrelor, *N* (%)	12 (20.0)	6 (13.95)	0.34**
ASA + prasugrel, *N* (%)	0 (0)	0 (0)	–
NOAC + clopidogrel, *N* (%)	1 (1.7)	0 (0)	0.51*
Vitamin K Antagonist + clopidogrel, *N* (%)	1 (1.7)	1 (2.3)	0.81*
Single Antiplatelet Therapy, *N* (%)	3 (5.0)	2 (4.65)	0.93*
Beta-blocker, *N* (%)	55 (91.7)	38 (88.4)	0.56**
Angiotensin-converting enzyme inhibitors/Angiotensin receptor blockers, *N* (%)	53 (88.3)	30 (69.8)	0.01**
Mineralocorticoid receptor antagonist, *N* (%)	9 (15.0)	9 (20.9)	0.43**
Diuretics, *N* (%)	22 (36.7)	11 (25.6)	0.23**
Statins, *N* (%)	55 (91.7)	43 (100)	0.05**

HDL, high-density lipoprotein; LDL, low-density lipoprotein.

* Results of Fisher test.

** Results of chi 2 test.

*** Results of *U*-Mann Whitney test.

**** Results of *t*-student test.

The number of patients who completed the first outpatient visit (6 weeks after AMI) was higher in the group with the APH-KOS app. Furthermore, the onsite time of the visit in this group was significantly shorter. Results are shown in Table 2.

**Table 2:** Results of AHP-KOS mobile app implementation.

Parameter	Active group (*n* = 60)	Reference group (*n* = 43)	*P* value
First outpatient visit (6 weeks post AMI) completed, *N* (%)	55 (91.7)	29 (67.4)	<0.001**
On-site visit duration time [min] (mean ± SD)	8.05 ± 3.30	11.33 ± 4.44	<0.001***

N, number; SD, standard deviation.

** Results of chi 2 test.

*** Results of *U*-Mann Whitney test.

In the further evaluation the relation between patients who have the best and worst adherence to using the AHP-KOS app by the means of the study aim was assessed. It was performed in order to investigate whether there is any particular type of patient characteristics, that would be most likely to follow with the adherence to the program. Analysis revealed that age was significantly associated with visit completion rates as presented in the Table 3. However, given the baseline age difference between groups it is unclear to strictly determine whether the observed differences in adherence were related only to the app intervention or to the technology adoption.

**Table 3:** Relationship between patient characteristics and adherence to the CR programme.

The first CR visit was completed (*n* = 84)			
Parameters	Active group (*n* = 55)	Control group (*n* = 29)	*P* -value
Age [years]	62.85 ± 8.28	69.4 ± 13.3	0.01**
Total cholesterol [mg/dl]	177.13 ± 45.18	164.96 ± 45.28	0.24**
LDL [mg/dl]	108.85 ± 42.89	103.94 ± 43.44	0.62**
HDL [mg/dl]	46.18 ± 17.29	42.31 ± 11.90	0.30***
Triglycerides [mg/dl]	136.60 ± 78.11	119.14 ± 55.68	0.47***
GFR [ml/kg/min]	78.50 ± 17.55	69.58 ± 23.40	0.17***
The first CR visit was not completed (*n* = 19)			
Parameters	Active group (*n* = 5)	Reference group (*n* = 14)	*P* -value
Age [years]	63.00 ± 6.55	72.64 ± 12.54	0.12**
Total cholesterol [mg/dl]	177.80 ± 39.58	172.21 ± 36.77	0.77**
LDL [mg/dl]	111.80 ± 43.71	111.28 ± 36.27	0.97**
HDL [mg/dl]	43.00 ± 15.26	38.85 ± 14.78	0.67***
Triglycerides [mg/dl]	114.00 ± 53.55	139.71 ± 63.01	0.48***
GFR [ml/kg/min]	79.80 ± 11.43	65.50 ± 24.54	0.28***

N, number; LDL, low density lipid cholesterol; HDL, high density lipid cholesterol; GFR, glomerular filtration rate; CR, cardiac rehabilitation.

** Results of *t*-student test.

*** Results of U-Mann–Whitney test.

## 6 Discussion

The MC-AMI complete cardiac rehabilitation program encompasses diagnostic procedures and suitable interventional therapy during the acute phase of MI, followed by coordinating visits, cardiac rehabilitation, and 4 outpatient visits within 12 months of follow-up (e.g., clinical evaluation and assessment for ICD/CRT implantation as a primary prevention of sudden cardiac death) (18). While these components represent the standard of care for MI survivors, research indicates that participation in MC-AMI leads to improved short-term and 1-year prognoses (8, 10). These positive outcomes were further validated in the analysis, which examined the largest cohort to date (comprising a total of 87,793 AMI patients enrolled between October 1, 2017, and December 31, 2018), with a follow-up period of up to 18 months (mean duration of 234 days) (19). On a population level, the study affirmed long-term prognostic benefits of MC-AMI (20). However, there are still concerns regarding different methods of improving patient adherence to the program itself. Comparing the number of patients included in the MC-AMI in 2022—only 27% of more than 70 thousand patients after AMI joined the program. As such, new approaches targeted to increase enrollment and adherence rates could further improve outcomes of the population after AMI.

The MC-AMI CR program itself consists of two different modules—stationary (hospitalization in the cardiac rehabilitation ward, eligible for higher risk patients, e.g., patients after cardiac arrest or elderly ones) and outpatient (everyday rehabilitation without hospital stay). Discharging physician decide to which variant of CR type is suitable. More specifically, the CR program is build with the principles of FITT (frequency, intensity, type and time) and divided into 3 models. Model A, designated for patients with low risk of cardiac arrest, with good exercise tolerance (MET >7, >100 Watts)—these patients are mostly given 60–90 min of exercises, such as: continuous endurance training on a cycle ergometer or treadmill (3–5 days/week), resistance training (3–5 days/week, 2–3 series) and set of general fitness exercises (5 days/week), to a max of 60%–80% of heart rate reserve (HRR). Model B is designated for patients with moderate and low risk, with >5MET or >75 Watts exercise tolerance, with lower duration (45–60 min/day) and intensity (50%–60% HRR). Model C is suitable for higher risk patients, with 3–5 MET or 50–75 Watts exercise tolerance, with even lower intensity (40%–50% HRR) and duration (max 45 min/day). Exercises are performed with supervision of rehabilitation specialists, both physicians and physiotherapists, while patients are instructed and monitored to maintain exercise level on 11–13/20 relative perceived effort Borg scale—this module is usually suited for 3 weeks. After, the patient is scheduled to the outpatient cardiology clinic for at least 3 visits throughout one year after AMI.

In hereby presented study we focused on the aspect of new ways of optimizing workflow as well as on the adherence to the program. As work burden in tertiary healthcare center remains high, such solutions could ease certain aspects of patient-oriented care. In our study, the designed AHP-KOS mobile app was developed as an assistant to clinicians working in outpatient clinic. Firstly, the app was responsible for gathering data regarding patient vital signs, followed by subjective health status assessment. Furthermore, before each scheduled visit, patient filled a questionnaire regarding basic cardiological medical history, especially these regarding most crucial: signs of exacerbation of heart failure or recurring angina symptoms that could implicate further need of invasive treatment (e.g., in patients with multivessel coronary disease or after several percutaneous coronary intervention, ergo—high risk patients). These options improved further visit process—as there is already data input in the hospital information system, which significantly reduces time needed to fill every certain point in patient medical history. On the other hand, as the AHP-KOS app works in two directions, patient can initiate contact with healthcare facility, especially in urgent clinical events, e.g., rapid onset of new angina—it could be done by additional visit or teleconsultation with physician. However, in our study group there was no need for such actions, yet it remains suitable for further use.

Based on the presented study results it can be assumed that the disparity in visit expenses between the two group favors the application of the AHP-KOS mobile app. By extrapolating this finding and considering the potential for additional visits per hour, the utilization of the AHP-KOS application indicates savings per standard working day. However, as healthcare practitioners, we acknowledge the inherent complexities and nuances involved in patient care, prioritizing quality and individual needs above all else. Nonetheless, it is conceivable that patients who have the means to leverage e-health solutions such as the mobile application discussed herein may experience enhancements in the delivery of medical services.

Previously, the use of the CR mobile app was described by Rivers et al. in 2022 addressing low participation rates in CR after AMI by the introduction of a mobile app-based CR program (Cardihab) for patients who declined conventional CR due to various constraints. Out of 204 patients offered CR following angioplasty, 99 obtained a conventional CR program (cohort 1), while 105 received an option of app-based CR in addition to conventional CR (cohort 2). The study found that CR participation significantly increased from 21% to 63% with the addition of the app, highlighting the potential of technology-based solutions to enhance CR uptake. However, some patients in cohort 2 declined both forms of CR, citing technology issues or other reasons such as frailty or comorbidities, suggesting the need for further innovation in CR delivery systems to address barriers to participation (21). Additionally, several meta-analyses and reviews shows that using mobile apps lead to better CR adherence among patients (22, 23). These findings correlate with the results of our study; therefore it is considerable that using CR mobile apps as described earlier is effective in the matter of adherence to the rehabilitation itself and could prove health-related benefits for patients after AMI.

In the aspect of coronary artery disease, Murphy et al. described a secondary preventive strategy with the use of the mobile app. The study systematically reviewed smartphone-based secondary prevention programs compared to traditional cardiac rehabilitation for patients with coronary artery disease. Analyzing 8 studies involving 1,120 patients across five countries, outcomes included a 6-minute walk test (6 MWT) distance, systolic blood pressure, LDL cholesterol, and body mass index (BMI). Results showed smartphone programs significantly improved exercise capacity measured by the 6 MWT, while other parameters like BMI reduction, systolic blood pressure, and LDL cholesterol levels showed no significant differences between smartphone and traditional rehabilitation groups (24).

Indraratna et al. in the systematic review and meta-analysis focused on mobile apps in managing chronic CVD, including CAD, heart failure, and hypertension. The review aimed to assess outcomes such as mortality, hospitalizations, blood pressure BP, and BMI. Through analysis of 26 randomized control trials (RCTs) involving 6,713 patients, and a meta-analysis of 12 RCTs, the study found that mobile app interventions were associated with a lower rate of hospitalizations in heart failure patients and a reduction in systolic blood pressure in patients with hypertension. These findings suggest the potential benefits of mobile phone interventions in managing chronic CVD and improving patient outcomes (25).

Any evaluation of the use of an application from the broader area of e-health requires consideration of multiple stakeholders in a holistic and integrated way. From a financial and management evaluation aspect, it requires an understanding of the potential benefits and costs that the application provides. The first step in measuring costs and benefits is to identify the relevant categories of costs and benefits affected by the e-health application. When it comes to the economic evaluation of the use of a specific information technology (including a mobile application), we need to choose an appropriate methodology and data collection strategy (26). In the context of the AHP-KOS mobile app, the presented solution enables hospitals to receive bonuses for effectively delivering coordinated medical care from the public payer through the timely provision of services throughout the defined period of outpatient and rehabilitation care. Additionally, it allows for the optimization of the work of medical and coordinating staff.

In the broader perspective, we can identify the various socio-economic benefits that these applications can provide (27). E-health involves complex management issues and competing options that require careful consideration of the expected benefits to outweigh the costs (28). Research in this area requires the use of appropriate criteria to evaluate such solutions. Mobile e-health applications have become very popular, yet however, the vast majority of these solutions, despite their good promise, face several challenges, including development, deployment, and maintenance efforts, lack of end-user acceptance, integration with other healthcare systems, difficulties in adapting to different users, and lack of adequate feedback to developers (29, 30). Additionally, the scalable maintenance of mobile applications incurs significant expenses, necessitating sustained revenue streams for developers to ensure continuous support. The volatile nature of the mobile application market facilitates rapid discontinuation of services. Consequently, there exists an imperative for legislative frameworks that secure equitable compensation for developers and assure enduring support for chosen applications, thereby facilitating their incorporation into established treatment protocols.

Furthermore, comprehensive economic evaluation helps e-health investment decisions and supports the long-term sustainability of e-health implementation (31). In the absence of robust evidence of effects, key decision-makers may doubt effectiveness, which in turn limits investment and long-term integration of eHealth services (32). Thus, it is important to identify the right enablers to facilitate the implementation of such solutions to achieve the intended vision and thus achieve better healthcare outcomes and move towards network-centric solutions. In this area, the implementation of process thinking in line with the lean management philosophy seems important. Lean is based on three main pillars: process optimization, patient-centered management, and employee engagement and leadership. Each of the three aspects must be implemented with equal attention (33). The implementation of e-health solutions in the lean e-health trend appears to be quite a challenge. On the one hand, we have to deal with the application of information technology and, on the other hand, with the appropriate process management that imposes the implementation of this technology. In doing so, we are mapping processes appropriately and organizing the internal structure of the healthcare entity (34, 35). Certainly, experiences in this area are growing and these experiences can be used in new development work in this area in the healthcare sector (36).

## 7 Limitations

This study regards patients from only one hospital in a particular region of Europe. Due to its nature, the study is not randomized. The analyzed application, due to legal constraints, was not fully integrated into the HIS system at the hospital where it was implemented. However, utilizing the application for automating patient interviews optimized visit time and served as a control for the substantive content of the interviews. Certainly, integrating the application with the IT system would allow for the automatic transmission of data related to appointment scheduling, interview content, and medical statistics in the future.

Another problem is that the patients using the app were significantly younger and more likely to be men. This is an important observation that indicates the possibility of using the described solutions rather in younger patients who can accept them. The results are promising but should be treated with caution in older patients. The requirement for smartphone ownership may have introduced selection bias, as patients without smartphones were systematically excluded from the intervention group. This may limit the generalizability of our findings to populations with lower smartphone adoption rates.

## 8 Conclusions

The obtained results indicate that the AHP-KOS mobile app among patients after myocardial infarction resulted in higher adherence to the cardiac rehabilitation program. Moreover, the implementation of the designed mobile app improved institutional workflow optimizing the duration of the visit (the possibility to perform more appointments) with a positive financial impact. It could be assumed that the AHP-KOS mobile app was more helpful for younger patients that older ones. The results presented in this article could provide data for further studies, regarding use of designed rehabilitation devices and software.

## Data Availability

The raw data supporting the conclusions of this article will be made available by the authors. In case of a data request it will be assessed and after verifying (with adjustment to the GDPR) the data will be shared.
