# Supplementary material for: Feasibility and efficacy of mobile app implementation among patients with acute myocardial infarction enrolled in coordinated cardiac rehabilitation program
Source: Front Digit Health. 2025 Nov 6;7:1606216. doi: 10.3389/fdgth.2025.1606216 (PMC12630128; doi:10.3389/fdgth.2025.1606216)
Supplement: Supplementary file 1 [file Datasheet1.pdf]

Supplementary Material. Pre-visit medical questionnaire for patients with the AHP-KOS mobile app.

Question Number	Question	Answers	Medical Interpretation
1	How do you rate your health compared to your previous visit/cardiological intervention?	- Better: overall improvement in health	Medical Status: Patient reports overall improvement in health
		- Worse: overall worsening of health	Medical Status: Patient reports overall worsening of health
		- Unchanged: no improvement/worsening of health	Medical Status: Patient reports no improvement/worsening of health
2	Do you experience shortness of breath every day?	- NO, I do not experience it even during daily activities, not even strenuous ones (e.g., vacuuming)	Medical Status: NYHA I
		- YES, I feel tired during daily strenuous activities	Medical Status: NYHA II
		- YES, I feel tired during minimal daily activities (going up 1 floor)	Medical Status: NYHA III
		- YES, tiredness even at rest	Medical Status: NYHA IV
3	Do you experience angina-like chest pains (similar to those before a heart attack)?	- Rather not, only occasionally during heavy, prolonged physical exertion	Medical Status: CCS I
		- YES, when going uphill (2 floors) and during long walks on flat ground	Medical Status: CCS II
		- YES, during minor physical exertion (going up 1 floor)	Medical Status: CCS III

Question Number	Question	Answers	Medical Interpretation
		- YES, even at rest	Medical Status: CCS IV
4	Do you frequently experience low blood pressure (below 105/80 mmHg) after a heart attack?	- YES	Medical Status: RR often <105/80mmHg
		- NO	Medical Status: RR; no hypotension
5	Do you frequently experience high blood pressure values (above 140/80 mmHg) after a heart attack?	- YES	Medical Status: Frequently RR above 140/80 mmHg
		- NO	Medical Status: RR; no spikes
6	Do you frequently experience low heart rate (below 60/min) after a heart attack?	- YES	Medical Status: HR: occasionally below 60/min at home according to patient's account
		- NO	Medical Status: HR; no low values at home according to patient's account
7	Do you frequently experience high heart rate (above 90/min) after a heart attack?	- YES	Medical Status: HR occasionally >90/min at home according to patient's account
		- NO	Medical Status: HR below 90/min at home according to patient's account

Question Number	Question	Answers	Medical Interpretation
8	Do you have leg swelling that occurred after a heart attack?	- YES	Medical Status: Patient reports swelling of lower limbs after MI
		- NO	Medical Status: Patient does not report swelling of lower limbs
9	Do you experience dizziness and/or fainting that occurred after a heart attack?	- YES	Medical Status: Patient complains of dizziness/fainting after MI
		- NO	Medical Status: Patient denies dizziness/fainting
10	Do you have a cough that occurred after a heart attack?	- YES	Medical Status: Patient complains of coughing after MI
		- NO	Medical Status: Patient denies coughing
11	Do you experience shortness of breath unrelated to physical exertion after a heart attack?	- YES	Medical Status: Patient reports shortness of breath unrelated to physical exertion after MI
		- NO	Medical Status: No resting dyspnea reported
12	Do you have symptoms of bleeding (e.g., black stools, red urine, nosebleeds) after a heart attack?	- YES	Medical Status: Patient reports symptoms of bleeding after MI
		- NO	Medical Status: Patient denies symptoms of bleeding (e.g., black stools, red urine, nosebleeds)

Question Number	Question	Answers	Medical Interpretation
13	Do you take your medication regularly?	- YES	Medical Status: Takes medication regularly
		- NO	Medical Status: Does not take medication regularly
14	Do you smoke cigarettes, including electronic cigarettes?	- NO	Medical Status: Still smoking cigarettes!!
		- YES	Medical Status: Does not smoke cigarettes
		- YES, occasionally	Medical Status: Patient reports still lighting up cigarettes!
